# Asthma Comorbidities: Frequency, Risk Factors, and Associated Burden in Children and Adolescents

**DOI:** 10.3390/children9071001

**Published:** 2022-07-03

**Authors:** Salvatore Fasola, Giuliana Ferrante, Giovanna Cilluffo, Velia Malizia, Pietro Alfano, Laura Montalbano, Giuseppina Cuttitta, Stefania La Grutta

**Affiliations:** 1 Institute of Translational Pharmacology, National Research Council, 90146 Palermo, Italy; velia.malizia@ift.cnr.it (V.M.); pietro.alfano@ift.cnr.it (P.A.); dr.montalbanolaura@gmail.com (L.M.); giuseppina.cuttitta@ift.cnr.it (G.C.); stefania.lagrutta@ift.cnr.it (S.L.G.); 2 Department of Surgical Sciences, Dentistry, Gynecology and Pediatrics, Pediatric Division, University of Verona, 37134 Verona, Italy; giuliana.ferrante@univr.it; 3 Department of Earth and Marine Sciences, University of Palermo, 90123 Palermo, Italy; giovanna.cilluffo@unipa.it

**Keywords:** asthma, adolescents, children, comorbidities, risk factors

## Abstract

Identifying asthma comorbidities in children is fundamental for improving disease management. We aimed to investigate the frequency of allergy-related comorbidities in children and adolescents with asthma, and to identify associated risk factors and disease burden. Between September 2015 and December 2018, 508 asthmatic patients (5–17 years) were consecutively enrolled. Parents answered a standardized questionnaire on the history of disease and risk factors. Comorbidities were classified based on the involvement of respiratory and/or extra-respiratory districts: asthma only (A, 13%), asthma with respiratory comorbidities (AR, 37%), asthma with extra-respiratory comorbidities (AER, 10%), and asthma with both respiratory and extra-respiratory comorbidities (ARER, 40%). Multinomial logistic regression showed that membership in the AR group was significantly associated with a maternal history of asthma (OR = 3.08, 95% CI: 1.23–7.72), breastfeeding ≥ three months (OR = 1.92, 1.06–3.46), early mold exposure (OR = 2.39, 1.12–5.11), and current environmental tobacco smoke exposure (OR = 2.06, 1.11–3.83). Membership in the AER group was significantly associated with the female gender (OR = 3.43, 1.54–7.68), breastfeeding ≥ three months (OR = 2.77, 1.23–6.22). ARER was significantly associated with all the aforementioned exposures. Patients with AR reported exacerbations in the last 12 months more frequently (*p* = 0.009). Several personal and environmental risk factors are associated with comorbidities in asthmatic children and adolescents, possibly worsening the disease burden.

## 1. Introduction

The study of comorbidities in asthmatic patients has not received the same level of attention compared to other chronic diseases, especially in pediatric age. However, there is increasing acknowledgment that asthma and multiple diseases often co-exist, leading to a significant impact on the overall health of patients [1].

Comorbidities in pediatric asthma include allergic diseases like rhinoconjunctivitis and eczema [2,3]. Other comorbidities encompass a range of conditions affecting the respiratory system like rhinosinusitis and sleep breathing disorders [4]. However, extra-respiratory disorders such as obesity have also been reported more frequently in children with asthma compared to healthy controls [5,6].

Overall, comorbidities pose a significant burden on individuals with asthma and the healthcare system, in terms of increased risk of exacerbations and high rates of hospitalizations, emergency department visits, and unscheduled doctor ambulatory care visits [7]. Recent works have studied the prevalence of allergy-related and other comorbidities, highlighting a higher burden concerning children and adolescents with asthma [8,9,10,11,12,13]. Hence, searching for comorbidity burdens in children with asthma is of paramount importance for more efficient care delivery.

Comorbidities can occur in asthmatic patients for different reasons. Notably, there is evidence that the co-existence of allergic comorbidities like rhinitis and eczema is significantly more frequent than in the general population, independently of Immunoglobulin (Ig) E sensitization [14]. This suggests that such asthma comorbidities share both genetic and environmental factors, which could be modifiable to a certain extent and thus could be considered for prevention strategies. However, even though the risk factors associated with pediatric asthma have been widely investigated, only a few studies evaluated their role in the estimation of asthma comorbidity burdens [15,16]. 

A better understanding of how risk factors may contribute to the asthma burden and the estimation of their role in asthma comorbidities may support clinicians in patient care and would be relevant in the research setting in order to develop intervention models for reducing asthma impact. Therefore, the knowledge of types of asthma comorbidity in childhood, and how such comorbidities could be addressed, accounting for the different role of risk factors, would be a desirable target for asthma care. 

Herein, we aimed to investigate the frequency of asthma-associated comorbidities in children and adolescents, and to identify associated risk factors and disease burden. In particular, we mainly focused on comorbidities correlating with allergy, as they are known to have a significant impact on the general health of patients of pediatric age.

## 2. Materials and Methods

### 2.1. Study Design and Population

**Figure 1:**
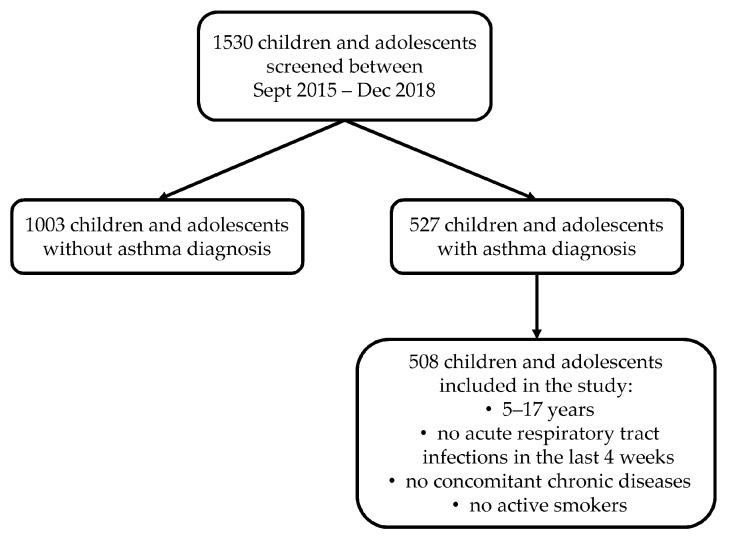
Flowchart describing the recruitment of individuals.

In this cross-sectional study, children and adolescents were consecutively recruited between September 2015 and December 2018 as a part of the ongoing CHildhood Asthma and Environment Research (CHASER) study, carried out at the outpatient clinic of the Pediatric Allergology & Pulmonology of Institute of Research and Biomedical Innovation–National Research Council (IRIB-CNR) of Palermo, Italy (Figure 1).

The inclusion criteria were: (1) doctor diagnosis of asthma according to the “Global Initiative for Asthma” (GINA) guidelines (www.ginasthma.org, accessed on 13 June 2022); (2) age 5–17 years; (3) ability to perform spirometry. The exclusion criteria were: (1) acute respiratory tract infections in the last four weeks; (2) concomitant chronic diseases (diabetes, congenital and genetic diseases, autoimmune and neuropsychiatric disorders); (3) active smoker. The study was approved by the local Institutional Ethics Committee (Palermo 1, Italy, No. 08/2014) and was registered on the central registration system ClinicalTrials.gov (accessed on 13 June 2022) (ID: NCT02433275). The study was carried out in compliance with Good Clinical Practice and in accordance with the Declaration of Helsinki. All of the participants were informed about all aspects concerned with the research and provided their consent before study entry.

### 2.2. Assessments

All children were clinically evaluated by well-trained physicians (VM, GF, and SLG) for the assessment of eligibility. Height (cm) and weight (kg) were measured in a standing position without shoes, using a stadiometer (Wunder HR1, Monza, Italy) and an electronic weighing scale (Seca, Hamburg, Germany), from which the body mass index (BMI) was derived (kg/m^2^). BMI standard deviation (SD) scores were derived based on the World Health Organization (WHO) age-specific reference values and were categorized as non-obese (less than or equal to 2 SD), and obese (greater than 2 SD).

Asthma diagnosis was performed according to the GINA guidelines. Skin prick tests were performed using a Stallerpoint-VC^®^ kit and a panel of seven aeroallergens (*Dermatophagoides* mix, *Alternaria alternata*, dog and cat dander, *Parietaria judaica*, grass pollen mix, olive pollen), plus positive (histamine 1%) and negative (saline) controls (Stallergènes Italia Srl., Milan, Italy). Allergens were pricked on the forearm, and reaction sizes were evaluated after 15 min. A positive reaction was defined as a skin response with a wheal ≥ 3 mm larger than the negative control test [17]. Children were categorized as non-sensitized (no positive reactions), mono-sensitized (one positive reaction), and poly-sensitized (>1 positive reactions).

Forced expiratory value in 1 s (FEV_1_) was measured using a portable spirometer (Pony FX, Cosmed, Rome, Italy) according to standardized guidelines [18]. The best of three acceptable and reproducible measurements was retained. FEV_1_ was expressed as a percentage of the predicted value [19]. Exhaled nitric oxide (eNO) was measured ‘off line’ using an electrochemical sensor (Hypair FeNO, Medisoft RAM, Italy). Air samples from the lower airways were continuously analyzed by the sensor at a flow rate of 50 mL/s, after fast inhalation maneuvers at total lung capacity. eNO was recorded as the mean of three measurements varying less than 10% [20]. Asthma severity and control level were assessed according to GINA guidelines (www.ginasthma.org, accessed on 13 June 2022). Symptom duration (years) and the number of exacerbations in the last 12 months were reported by the parents.

All of the parents were interviewed using a modified version of the structured SIDRIA (Italian Studies on Respiratory Disorders in Children and the Environment) questionnaire [21], including questions about the history of diseases and early (first year of life) and current (last 12 months) exposure to host and environmental risk factors. 

A subset of children and adolescents providing their consent for a subjective symptom assessment filled a Visual Analog Scale (VAS), the Childhood Asthma Control Test (C-ACT, for children aged 6–11), and the Asthma Control Test (ACT, for adolescents aged 12–17), the Pediatric Asthma Quality of Life Questionnaire (PAQLQ), and the Pittsburgh Sleep Quality Index (PSQI).

### 2.3. Asthma Comorbidities

Comorbidities were classified according to the involvement of respiratory and/or extra-respiratory districts (Table 1): asthma only (A), asthma with respiratory comorbidities (AR: rhinitis OR sinusitis OR snoring), asthma with extra-respiratory comorbidities (AER: food allergy OR gastroesophageal reflux OR eczema OR urticaria OR angioedema OR anaphylaxis), asthma with both respiratory and extra-respiratory comorbidities (ARER). Each comorbidity was defined as a positive answer to a specific question of the form: “Has your child ever been diagnosed with [*name of the disease*] by a doctor?”.

### 2.4. Host and Environmental Risk Factors

The questionnaire included questions about maternal history of asthma and allergic diseases (rhinitis, eczema, atopy), parent education (categorized as lower than or at least eight years), mode of delivery (vaginal/cesarean), prematurity (gestational age lower than 37 weeks), and feeding practices (categorized as exclusive breastfeeding for at least three months after birth or not). We also recorded current/early exposure to environmental tobacco smoke (ETS, mother and/or father), exposure to ETS during pregnancy (mother), current/early exposure to pets (dog/cat), current/early exposure to molds (mold/dampness/fungi on the walls or on the ceiling in the child’s bedroom), and current traffic exposure (residential proximity to a high-traffic road).

### 2.5. Visual Analog Scale

VAS quantifies the subjective feeling of well-being. This scale ranges from 0 (poor well-being) to 10 (high well-being). Patients were asked to place a cross on a 10 cm line to indicate their perception of well-being [22].

### 2.6. Childhood Asthma Control Test and Asthma Control Test

The C-ACT is a validated questionnaire for children aged 4–11 years, including seven items related to the last four weeks. The first four items, answered by the child, refer to the perception of asthma control, limitation of activities, coughing, and night awakenings. The other three items, answered by the caregiver, refer to daytime complaints, wheezing, and night awakenings. The total C-ACT score ranges from 0 (poor control) to 27 (optimal control) [23]. 

The ACT is a validated questionnaire for children aged ≥ 12 years, including five items related to the last four weeks. The five items, completed by the patient, refer to the perception of asthma control, limitation of activities, coughing, and night awakenings. The total ACT score ranges from 5 (poor control) to 25 (optimal control) [24].

### 2.7. Pediatric Asthma Quality of Life Questionnaire

The PAQLQ investigates the quality of life in asthmatic children during the last week. It includes three domains about symptoms, activity limitations, and emotional function. Questions are answered by the patients, and the total score ranges from 1 (poor quality of life) to 7 (good quality of life) [25].

### 2.8. Pittsburgh Sleep Quality Index

The PSQI is a self-administered questionnaire based on a four-week recall that includes 19 questions in seven domains: subjective sleep quality, sleep latency, sleep duration, habitual sleep efficiency, sleep disturbance, use of sleep medications, and daytime dysfunction. The total score ranges from 0 (good sleep quality) to 21 (poor sleep quality) [26].

### 2.9. Statistical Analyses

The distribution of risk factors and indicators of disease burden was compared among the four comorbidity groups using the Kruskal-Wallis test for quantitative variables and the χ^2^ test for categorical variables. For children and adolescents providing their consent for symptom assessment, the distributions of VAS, C-ACT/ACT, PAQLQ, and PSQI by comorbidity group were visually displayed using boxplots.

The risk factors associated with a *p*-value lower than 0.20 were included in a multivariable, multinomial logistic regression analysis using membership in the comorbidity groups as the categorical outcome and A as the reference group. A parsimonious model was obtained through a stepwise approach using the Akaike information criterion (AIC) as a goodness-of-fit statistic for model comparison (the lower the better). Sensitivity analyses were carried out in the final model by replacing “Breastfeeding ≥ three months” with “Breastfeeding ≥ six months” or “Breastfeeding ≥ 12 months”, and replacing BMI with BMI SD scores or obesity. The model associated with the best AIC was retained.

Associations were reported as odds ratios (OR) and corresponding 95% confidence intervals (CI) and were visually displayed for each comorbidity group. The statistical analyses were performed through R version 4.0.2 (R Foundation for Statistical Computing, Vienna, Austria). Statistical significance was set at *p* < 0.05.

## 3. Results

Out of 1530 screened children and adolescents, 527 received an asthma diagnosis and 508 met the other inclusion criteria: 68 (13%) were in A, 188 (37%) were in AR, 50 (10%) were in AER and 202 (40%) were in ARER (Table 2).

Group membership was significantly associated with gender (more females in the AER group, *p* = 0.014), BMI (higher in the AR group, *p* = 0.014), asthma duration (longer in the ARER group, *p* = 0.041), allergic sensitization (more frequent in the AR and ARER groups, *p* = 0.036), maternal history of asthma, (more frequent in the AR and ARER groups, *p* = 0.015), and breastfeeding ≥ three months (more frequent in the AR, AER and ARER groups, *p* = 0.031). Weaker associations (*p* < 0.20) were found with age, maternal histories of allergies, early pet exposure, early mold exposure, and current ETS exposure.

**Figure 2:**
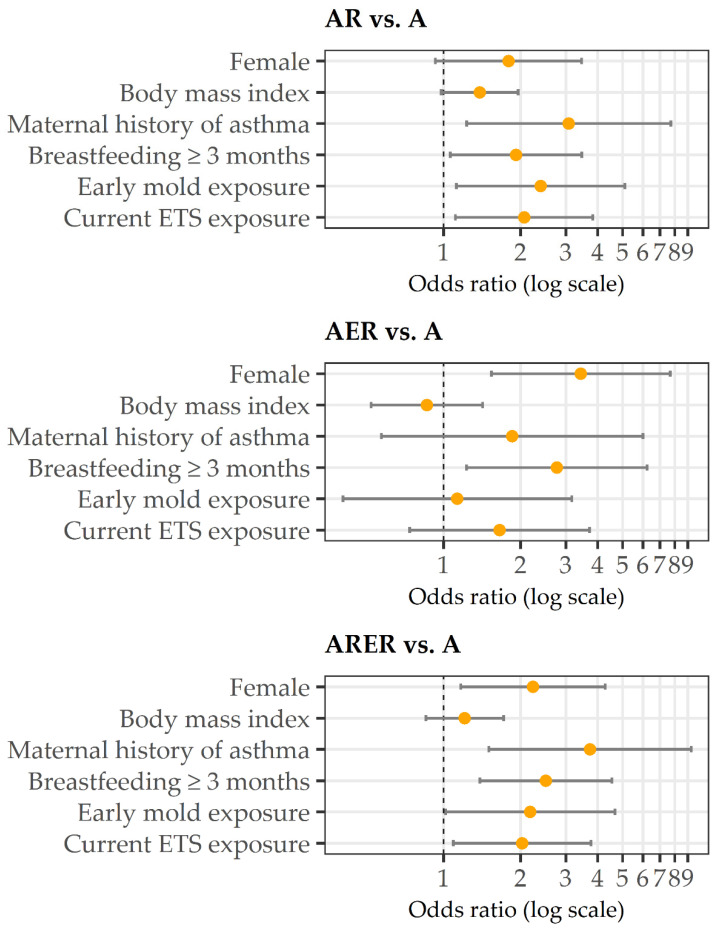
Estimated odds ratios (orange points) and 95% confidence intervals (bars) from the multinomial logistic regression model. A: asthma only. AR: asthma with respiratory comorbidities. AER: asthma with extra-respiratory comorbidities. ARER: asthma with both respiratory and extra-respiratory comorbidities. ETS: Environmental Tobacco Exposure.

The covariates selected by the stepwise approach as independent determinants of increased comorbidities risk are reported in Table 3 and Figure 2. The model including “Breastfeeding ≥ three months” fitted better (AIC = 1239.926) than the models including “Breastfeeding ≥ six months” and “Breastfeeding ≥ 12 months” (AIC = 1239.926 and AIC= 1241.406, respectively). The model including BMI fitted better (AIC = 1239.926) than the models including BMI SD scores or obesity (AIC= 1243.278 and AIC= 1241.139, respectively). 

Membership in the AR rather than in the A group was significantly associated with a maternal history of asthma (OR = 3.08), breastfeeding ≥ three months (OR = 1.92), early mold exposure (OR = 2.39), and current ETS exposure (OR = 2.06). 

Membership in the AER rather than in the A group was significantly associated with the female gender (OR = 3.43) and breastfeeding ≥three months (OR = 2.77). 

ARER status was consistently significantly associated with female gender (OR = 2.23), maternal history of asthma (OR = 3.73), breastfeeding ≥three months (OR = 2.50), early mold exposure (OR = 2.18), and current ETS exposure (OR = 2.03).

No significant differences were found between the groups in terms of indicators of disease burden, except for the number of exacerbations during the last 12 months, which was significantly higher in the AR and ARER groups (*p* = 0.009) (Table 4).

**Figure 3:**
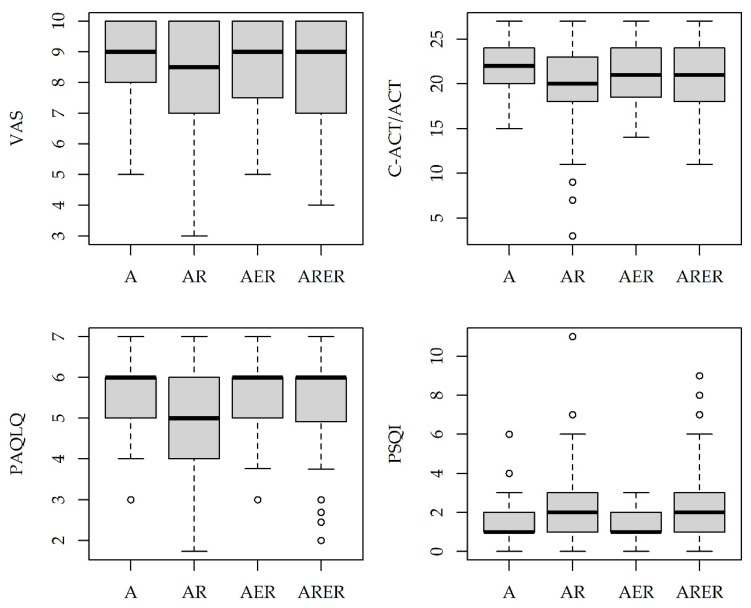
Distribution of VAS, CACT/ACT, PAQLQ, and PSQI scores by group in 225 children/adolescents who completed the questionnaires. Boxplots represent the median (central line), 25th–75th percentiles (box), and min-max non-outlier values (whiskers). A: asthma only. AR: asthma with respiratory comorbidities. AER: asthma with extra-respiratory comorbidities. ARER: asthma with both respiratory and extra-respiratory comorbidities.

A subset of 225 subjects (15% A; 35% AR; 12% AER; 38% ARER) completed the VAS, C-ACT/ACT, PAQLQ, and PSQI questionnaires. For each tool, global scores in the four groups are represented in Figure 3. A significant association was only found for PSQI, which was higher in the AR and ARER groups (*p* = 0.034, Table 4).

## 4. Discussion

The current study provided new insights into the characteristics of asthma comorbidities in children, focusing on their risk factors and their impact on disease burden. Out of the 508 included patients, 13% had A, 37% had AR, 10% had AER and 40% had ARER. We found that female gender, maternal history of asthma, breastfeeding, early mold exposure, and current ETS exposure were significant risk factors for asthma comorbidities. We also observed more exacerbations and a lower sleep quality in children with respiratory comorbidities.

We found a significantly higher risk of extra-respiratory comorbidities (AER and ARER) in females. Sex differences in asthma prevalence may be ascribed to sex hormones and the interaction of socioeconomic factors, access to resources (such as nutrition and air quality), comorbidities, and healthcare in developing/developed countries [27]. Furthermore, a previous study showed that asthma, allergic rhinitis, atopic dermatitis, and allergic conjunctivitis have different prevalences and are associated with different risk factors in infancy. Among boys, allergies are more frequent in childhood; this rapidly changes during girls’ sexual development, with a lifelong predominance of allergic diseases in females. Such a finding might be ascribed to the influence of sex hormones, differences in lifestyles, diet, and adherence to treatment, but it deserves to be further investigated [28].

A significantly higher BMI was observed in children with respiratory comorbidities (AR and ARER) (Table 2). In the multivariable regression model, BMI was indeed retained by the stepwise selection approach, even if its effect was not significant. Indeed, respiratory disorders like allergic rhinitis [29] and snoring [30] have been more frequently reported in overweight/obese children with asthma than in those with normal weight. Overall, these findings underline the importance of addressing strategies for weight management in children with asthma who are overweight/obese, with the aim of reducing the risk for additional comorbidities that can have short and long-term health consequences. 

We observed that maternal history of asthma had an important role in the risk of respiratory comorbidities (AR and ARER) for the offspring. Previous research found that maternal asthma is significantly associated with an increased prevalence of asthma in children [31]. In particular, there is evidence that maternal asthma during pregnancy is significantly associated with an increased prevalence of asthma in offspring [32]. Similarly, Martel et al. found that maternal history of asthma during pregnancy is associated with an increased incidence of asthma in their children, along with comorbidities like wheezing [33]. Conversely, the effect of maternal history of allergies was not statistically significant, as was the case in previous birth cohort studies [8,34]. However, it should be pointed out that these studies investigated the role of parental allergy on the risk of developing allergic multimorbidity in their children, instead of the maternal one alone. Therefore, the role of maternal history of allergy on the risk of respiratory comorbidities for the offspring needs to be further elucidated.

Our data showed that exclusive breastfeeding for at least three months was a risk factor for respiratory and/or extra-respiratory comorbidities. To date, there is no clear evidence of breastfeeding’s protective effect against single allergic disorders (i.e., eczema, food allergy, and rhinitis), and there is low-grade quality evidence about the association between breastfeeding duration (>three to four months) and the reduced risk of asthma in children and adolescents [35,36]. Therefore, the role of human milk in the prevention of allergic diseases remains controversial. This may be ascribed to the heterogeneity of study populations and outcome definitions, and to the lack of randomized controlled trials reporting detailed information on the maternal diet during breastfeeding. As a matter of fact, while many studies endorse a protective effect, other studies suggest that breastfeeding may promote allergies [35]. Nonetheless, our results are consistent with the speculation that the milk of atopic or asthmatic mothers (up to 40% in our comorbidity groups) may differ in terms of immunologically active substances, so that breastfeeding in these populations may have a negative effect on the risk of developing asthma comorbidities [37]. 

Early mold exposure and current ETS exposure were also found to be significantly associated with a higher risk of respiratory comorbidities (AR and ARER). This result is not surprising, given that domestic exposure to mold/dampness and passive smoke exposure have both been associated with allergic respiratory disorders like asthma and rhinitis [3], as well as rhinitis-asthma comorbidity [38]. 

We observed significantly higher percentages of allergic poly-sensitizations in the AR and ARER groups (respiratory comorbidities) (Table 2), which is consistent with previous studies demonstrating the association between atopy and asthma symptoms in pediatric age [39,40,41]. Nevertheless, allergic sensitization did not enter the multivariable regression model after the stepwise covariate selection, suggesting that factors other than allergy (i.e., the aforementioned host and environmental risk factors) may be associated with multimorbidity in children with asthma.

Comorbidities are increasingly recognized as potential contributors to uncontrolled asthma [42]. Although we did not find significant differences in terms of asthma control among the comorbidity groups, we found an increased number of exacerbations during the last 12 months in the AR group, confirming that asthma control could be significantly impaired by the presence of respiratory comorbidities [43]. In particular, rhinitis is often associated with asthma, and it can be responsible for lower C-ACT scores [44]. We found consistently lower C-ACT/ACT scores in AR than in A. Moreover, in our study, subjects with respiratory comorbidities showed higher PSQI scores, which is in line with a previous study where a trend toward increased sleep disturbance was observed in asthmatic patients (32% children) with allergic rhinitis and sinusitis [43]. 

The main strength of this study is to have collected both subjective and objective information. Indeed, medical histories based on questionnaire data may be affected by recall bias. In particular, some uncertainty might have affected parental reports of events that occurred during the early life of the child. However, the use of objective tools, like spirometry and the skin prick test, allowed for the enhancing of the clinical assessment of the study population. A possible limitation is related to the retrospective cross-sectional nature of the study, therefore caution must be used when generalizing these findings to other populations of children with asthma.

## 5. Conclusions

The current study provided new insights into the frequency, risk factors, and burden associated with asthma comorbidities in pediatric age. Limiting the exposure to avoidable environmental risk factors, starting from early life, may help reduce respiratory comorbidities and the associated burden, mainly in terms of exacerbations and sleep disturbance. Further longitudinal studies could be useful in increasing knowledge about the role of risk factors in childhood asthma comorbidities.

## Figures and Tables

**Table 1:** Definition of the comorbidity groups. A: asthma only. AR: asthma with respiratory comorbidities. AER: asthma with extra-respiratory comorbidities. ARER: asthma with both respiratory and extra-respiratory comorbidities.

Reference Group	
A	asthma only
**Comorbidity Group**	
AR	asthma AND (rhinitis OR sinusitis OR snoring)
AER	asthma AND (food allergy OR gastroesophageal reflux OR eczema OR urticaria OR angioedema OR anaphylaxis)
ARER	asthma AND (rhinitis OR sinusitis OR snoring) AND (food allergy OR gastroesophageal reflux OR eczema OR urticaria OR angioedema OR anaphylaxis)

**Table 2:** Subject characteristics by comorbidity group.

	Overall *n* = 508 (100%)	A *n* = 68 (13%)	AR *n* = 188 (37%)	AER *n* = 50 (10%)	ARER *n* = 202 (40%)	*p*-Value
**Host Factors**						
Female gender	181 (36)	17 (25)	61 (32)	26 (52)	77 (38)	**0.014**
Age, years	8.6 (2.8)	8.1 (2.9)	9.0 (2.8)	8.3 (2.6)	8.6 (2.8)	*0.064*
Body mass index, kg/m^2^	19.4 (4.3) ^1^	18.9 (4.5)	20.0 (4.4)	18.1 (4.1)	19.4 (4.2)	**0.014**
Asthma duration, years	3.8 (2.3)	3.5 (1.9)	3.7 (2.6)	3.7 (1.5)	4.0 (2.3)	**0.041**
Allergic sensitization						**0.036**
Non-sensitized	123 (24)	25 (37)	39 (21)	11 (22)	48 (24)	
Mono-sensitized	143 (28)	18 (26)	50 (27)	21 (42)	54 (27)	
Poly-sensitized	242 (48)	25 (37)	99 (53)	18 (36)	100 (50)	
Parent education ≥ eight years	447 (88)	57 (84)	168 (89)	42 (84)	180 (89)	0.485
Maternal history of asthma	107 (21)	6 (9)	42 (22)	7 (14)	52 (26)	**0.015**
Maternal history of allergies	185 (36)	17 (25)	74 (39)	15 (30)	79 (39)	*0.109*
Cesarean delivery	285 (56)	38 (56)	110 (59)	32 (64)	105 (52)	0.376
Preterm birth	57 (11)	7 (10)	28 (15)	3 (6)	19 (9)	0.200
Breastfeeding ≥ three months	331 (65)	35 (51)	119 (63)	36 (72)	141 (70)	**0.031**
**Environmental Factors**						
Early pet exposure	58 (11)	3 (4)	27 (14)	4 (8)	24 (12)	*0.137*
Current pet exposure	104 (20)	12 (18)	46 (24)	9 (18)	37 (18)	0.401
Early mold exposure	126 (25)	10 (15)	54 (29)	8 (16)	54 (27)	*0.052*
Current mold exposure	102 (20)	11 (16)	35 (19)	7 (14)	49 (24)	0.236
ETS exposure during pregnancy	199 (39)	25 (37)	76 (40)	15 (30)	83 (41)	0.500
Early ETS exposure	189 (37)	26 (38)	71 (38)	15 (30)	77 (38)	0.744
Current ETS exposure	210 (41)	21 (31)	85 (45)	18 (36)	86 (43)	*0.176*
Current traffic exposure	151 (30)	15 (22)	59 (31)	12 (24)	65 (32)	0.317

^1^ 128 children (25%) were obese. Data are presented as *n* (%) or mean (SD). A: asthma only. AR: asthma with respiratory comorbidities. AER: asthma with extra-respiratory comorbidities. ARER: asthma with both respiratory and extra-respiratory comorbidities. ETS: Environmental Tobacco Exposure. Early: first year of life. Current: last 12 months. Significant *p*-values are in bold. *p*-values lower than 0.20 are in italics.

**Table 3:** Estimated odds ratios (OR), *p*-values and 95% confidence intervals (CI) from the multinomial logistic regression model.

	AR vs. A		AER vs. A		ARER vs. A	
	OR (*p*-Value)	95% CI	OR (*p*-Value)	95% CI	OR (*p*-Value)	95% CI
Female	1.79 (0.083)	0.93–3.46	3.43 (**0.003**)	**1.54–7.68**	2.23 (**0.015**)	**1.17–4.28**
Body mass index (five-unit increase)	1.38 (0.066)	0.98–1.95	0.86 (0.558)	0.52–1.42	1.21 (0.288)	0.85–1.71
Maternal history of asthma	3.08 (**0.016**)	**1.23–7.72**	1.85 (0.305)	0.57–6.00	3.73 (**0.005**)	**1.50–9.27**
Breastfeeding ≥ three months	1.92 (**0.031**)	**1.06–3.46**	2.77 (**0.014**)	**1.23–6.22**	2.50 (**0.002**)	**1.38–4.54**
Early mold exposure	2.39 (**0.024**)	**1.12–5.11**	1.13 (0.816)	0.40–3.17	2.18 (**0.045**)	**1.02–4.66**
Current ETS exposure	2.06 (**0.022**)	**1.11–3.83**	1.65 (0.224)	0.74–3.71	2.03 (**0.025**)	**1.09–3.76**

A: asthma only. AR: asthma with respiratory comorbidities. AER: asthma with extra-respiratory comorbidities. ARER: asthma with both respiratory and extra-respiratory comorbidities. ETS: Environmental Tobacco Exposure. Significant associations are in bold.

**Table 4:** Indicators of disease burden by comorbidity group.

	Overall *n* = 508 (100%)	A *n* = 68 (13%)	AR *n* = 188 (37%)	AER *n* = 50 (10%)	ARER *n* = 202 (40%)	*p*-Value
Asthma severity						0.666
Intermittent	246 (48)	32 (47)	93 (49)	25 (50)	96 (48)	
Mild persistent	170 (33)	27 (40)	65 (35)	15 (30)	63 (31)	
Moderate/severe persistent	92 (18)	9 (13)	30 (16)	10 (20)	43 (21)	
Asthma control						0.220
Well controlled	195 (38)	34 (50)	64 (34)	21 (42)	76 (38)	
Partly controlled	107 (21)	8 (12)	46 (24)	8 (16)	45 (22)	
Uncontrolled	206 (41)	26 (38)	78 (41)	21 (42)	81 (40)	
Exacerbations last 12 months	2.6 (3.4)	2.0 (3.2)	3.0 (3.4)	2.2 (3.4)	2.6 (3.3)	**0.009**
FEV_1_ % predicted	96.5 (13.2)	95.9 (14.6)	96.2 (12.0)	93.8 (13.3)	97.8 (13.7)	0.193
eNO, ppb	13.2 (10.1)	13.0 (8.9)	14.9 (11.9)	11.5 (7.4)	12.2 (9.1)	0.185
C-ACT/ACT ^1^	20.6 (4.1)	21.8 (2.7)	19.7 (4.8)	20.9 (3.7)	20.8 (3.8)	0.215
PSQI ^1^	2.1 (1.9)	1.5 (1.3)	2.3 (1.8)	1.4 (0.8)	2.3 (2.2)	**0.034**
PAQLQ ^1^	5.3 (1.2)	5.5 (1.0)	5.2 (1.2)	5.4 (1.1)	5.4 (1.2)	0.635
VAS ^1^	8.3 (1.8)	8.7 (1.5)	8.1 (1.9)	8.5 (1.8)	8.2 (1.7)	0.404

Data are presented as *n* (%) or mean (SD). A: asthma only. AR: asthma with respiratory comorbidities. AER: asthma with extra-respiratory comorbidities. ARER: asthma with both respiratory and extra-respiratory comorbidities. FEV_1_: forced expiratory volume in 1 s. eNO: exhaled nitric oxide. Significant *p*-values are in bold. ^1^ Available for 225 subjects providing their consent for symptom assessment.

## Data Availability

The data that support the findings of this study are available from the corresponding author upon reasonable request.
